# Development of a dedicated Golden Gate Assembly Platform (RtGGA) for *Rhodotorula toruloides*

**DOI:** 10.1016/j.mec.2022.e00200

**Published:** 2022-05-23

**Authors:** Nemailla Bonturi, Marina Julio Pinheiro, Paola Monteiro de Oliveira, Eka Rusadze, Tobias Eichinger, Gintare Liudžiūtė, Juliano Sabedotti De Biaggi, Age Brauer, Maido Remm, Everson Alves Miranda, Rodrigo Ledesma-Amaro, Petri-Jaan Lahtvee

**Affiliations:** a Institute of Technology, University of Tartu, Tartu, Estonia; b Department of Chemistry and Biotechnology, Tallinn University of Technology, Tallinn, Estonia; c Department of Materials and Bioprocess Engineering, School of Chemical Engineering, University of Campinas, Campinas, Brazil; d Institute of Molecular and Cell Biology, University of Tartu, Tartu, Estonia; e Imperial College Centre for Synthetic Biology, Imperial College London, London, UK; f Department of Bioengineering, Imperial College London, London, UK

**Keywords:** *Rhodotorula toruloides*, Synthetic biology, Metabolic engineering, Non-conventional yeast, Oleaginous yeast, Golden gate assembly

## Abstract

*Rhodotorula toruloides* is a potential chassis for microbial cell factories as this yeast can metabolise different substrates into a diverse range of natural products, but the lack of efficient synthetic biology tools hinders its applicability. In this study, the modular, versatile and efficient Golden Gate DNA assembly system (RtGGA) was adapted to the first basidiomycete, an oleaginous yeast *R. toruloides*. *R. toruloides* CCT 0783 was sequenced, and used for the GGA design. The DNA fragments were assembled with predesigned 4-nt overhangs and a library of standardized parts was created containing promoters, genes, terminators, insertional regions, and resistance genes. The library was combined to create cassettes for the characterization of promoters strength and to overexpress the carotenoid production pathway. A variety of reagents, plasmids, and strategies were used and the RtGGA proved to be robust. The RtGGA was used to build three versions of the carotenoid overexpression cassette by using different promoter combinations. The cassettes were transformed into *R. toruloides* and the three new strains were characterized. Total carotenoid concentration increased by 41%. The dedicated GGA platform fills a gap in the advanced genome engineering toolkit for *R. toruloides*, enabling the efficient design of complex metabolic pathways.

## Highlights

• *R. toruloides* CCT 0783 was sequenced, and it is the biggest genome reported for this species.
• A dedicated Golden Gate Assembly platform (RtGGA) was adapted to *R. toruloides*.
• RtGGA is the first dedicated platform for a basidiomycete.
• Different reagents and approaches for assemblies were used and RtGGA found to be robust.
• RtGGA was used for building a pathway for overexpression of carotenoids and titers improved by 41%.

## 1 Introduction

The Sustainable Development Goals (SDGs) of the United Nations (UN) mark its historic shift towards the integration of economic and social development with environmental sustainability. Promoting food security and improved nutrition, ensuring sustainable use of resources and production patterns, and taking urgent action to combat climate change are among its directives (United Nations General Assembly, 2015). Microbial cell factories are an essential element for meeting UN's SDGs as biofuels, pharmaceuticals, and other chemicals can be produced from sustainable raw materials using engineered or native organisms. Microbial cell factories do not compete with food and arable land resources, they are not directly affected by different locations or climates (Koutinas et al., 2014) and they can use lignocellulosic biomass hydrolysates and biological waste as substrates. Being able to use such substrates is interesting, due to their abundance, low cost, and sustainability (Adrio, 2017).

Strain development is a crucial part of improving titers, yields, and rates when using microbial cell factories and this work is typically onerous (involves millions of dollars), takes 3–10 years to complete (Opgenorth et al., 2019), and requires several loops of the Design-Build-Test-Learn (DBTL) cycle (Nielsen and Keasling, 2016). Developing versatile, fast, standardized, and modular tools for building pathways are necessary for obtaining optimized strains using the DBTL cycle (Larroude et al., 2019). There has been an advancement in synthetic biology in the past decade that has led to the creation of several robust gene assembly platforms that can be exploited for metabolic engineering and pathway optimization, such as Sequence and Ligation-Independent Cloning (SLIC) (Li and Elledge, 2007), Gibson assembly (GA) (Gibson et al., 2009), Circular Polymerase Extension Cloning (CPEC) (Quan and Tian, 2011), and Golden Gate assembly (GGA) (Engler et al., 2008, 2009).

GGA provides a way to construct, diversify, and optimize multi-gene pathways exploiting the ability of Type IIS restriction endonucleases, such as BsaI, to cleave outside of their recognition sequence. The recognition sites are strategically placed distal to the cleavage site of inserts and cloning vectors, and, as such, the endonucleases can remove the recognition site from the assembly leaving 4 nucleotides long overhangs. As the overhang sequence is not dictated by the endonuclease, no scar sequence is introduced, and the specificity of each overhang allows simultaneous orderly assembly of multiple fragments. Digestion and ligation can be carried out at the same time when combining a DNA ligase with the endonucleases (Engler et al., 2008, 2009), therefore, several DNA fragments can be seamlessly assembled in the desired order in a one-pot reaction (Weber et al., 2011).

The Yeast Toolkit (YTK) is based on GGA and relies on an organizational system of up to three tiers of plasmids for storage or use (Lee et al., 2015). Parts, such as promoters, genes, terminators, etc., are stored in level 1 plasmids, called parts library. At level 2, level 1 promoter, gene, and terminators are assembled to create transcriptional units (TUs). Multiple TUs and other parts (integration sites and markers) are assembled at level 3 into a multi-gene vector. The standardization of DNA fragments by position-specific 4 nt overhangs offers the opportunity to shuffle around parts in a one-pot reaction, making the diversification and optimization of engineered pathways a less laborious affair. YTK was developed for the most traditional yeast cell factory, *Saccharomyces cerevisiae*, but it has been expanded to other microbial cell factories, such as *Yarrowia lipolytica* (Celińska et al., 2017; Larroude et al., 2019), *Pichia pastoris* (Prielhofer et al., 2017), *Kluyveromyces marxianus* (Rajkumar et al., 2019), and *Ashbya gossypii* (Ledesma-Amaro et al., 2018).

Among the microbial cell factories, the nonconventional yeast *Rhodotorula toruloides* is considered a promising biotech workhorse (Park et al., 2017), as it can consume a variety of carbon and nitrogen sources (Lopes et al., 2020a; Xu and Liu, 2017), has an improved tolerance towards inhibitors present in lignocellulosic hydrolyzates (Bonturi et al., 2017a), and can naturally co-produce industrially important molecules (lipids, carotenoids, and enzymes) (Park et al., 2017). Microbial lipids can be considered potential feedstock for oleochemicals and applied in different products, such as biofuels, cosmetics, plastics, coatings, surfactants, lubricants, paints, among others (Adrio, 2017). Carotenoids are used for food and pharmaceutical industries as A-vitamin precursors, colourants, or antioxidants. *R. toruloides* produces mainly γ-carotene, β-carotene, torulene, and torularhodin (Mata-Gómez et al., 2018). Today, torulene and torularhodin are not as commercially important as β-carotene, but due to their valuable antioxidant properties, the interest in their production has been increasing lately (Kot et al., 2018).

In the past decade, omics studies shed light on the genome and metabolic pathways of *R. toruloides* (Hu and Ji, 2016; Kumar et al., 2012; Morin et al., 2014; Zhu et al., 2012), and its modification was made possible by the identification of constitutive (Nora et al., 2019; Liu et al., 2016; Wang et al., 2016) and inducible promoters (Johns et al., 2016; Liu et al., 2015), and selectable markers based in auxotrophies (Yang et al., 2008) and antibiotics (Lin et al., 2014). Despite these advances, there is still a lack of a high throughput DNA assembly platform for this yeast. This is attributed to several factors. *R. toruloides* genes have a very high GC content (∼62%) (Sambles et al., 2017a), which hinders PCR reactions due to disruption polymerase efficiency and accuracy, hinders gene synthesis, and the design specific primers without reaching an extremely high annealing temperature, and heterologous genes need to be codon-optimized (Lin et al., 2014). Also, there is a lack of known autonomously replicating sequence (ARS) elements, therefore metabolic engineering requires the integration of genes and pathways into the genome (Tsai et al., 2017). Furthermore, integration cassettes require long homology arms (500–1000 bp), as the non-homologous end joining (NHEJ) is the default mechanism of DNA repair in *R. toruloides*. Due to the latter, deleting the gene KU70, part of the main component of NHEJ, improves gene deletion and integration frequency without negatively affecting the oleaginous and fast-growing features of *R. toruloides* (Koh et al., 2014).

A crucial part of utilizing *R. toruloides* as a microbial cell factory is the ability to engineer the metabolic pathways in the cell in an efficient and standardized manner (Park et al., 2017). The current work aimed at the development of a YTK-inspired *R. toruloides* Golden Gate dedicated assembly (RtGGA) platform. For this purpose, we have: (i) sequenced the genome of *R. toruloides* CCT0783, which is the parental strain of CCT7815, a tolerant strain used in several works using lignocellulosic hydrolysates as carbon sources (Bonturi et al., 2017a; Lopes et al., 2020b; Pinheiro et al., 2020); (ii) characterized six native promoters; and (iii) as the proof of concept of such a platform, simultaneous deletion of KU70 (for improving future deletions/integrations efficiencies) and overexpression of three native genes in the carotenoid biosynthesis pathway – geranylgeranyl diphosphate synthase (*crtE*, EC 2.5.1.29), phytoene dehydrogenase (*crtI*, EC 1.3.99.30), and phytoene synthase/lycopene cyclase (*crtYB*, EC 2.5.1.32/EC 5.5.1.19) – was carried out. Once this platform was established, it was used for the assembly of different constructs to the metabolic engineering of *R. toruloides* for further improvement of the carotenoid production using xylose, the main sugar of hemicellulosic biomass hydrolysates.

## 2 Materials and methods

### 2.1 Microorganism, genomic DNA extraction, sequencing, analysis, and annotation

The strain *R. toruloides* CCT 0783 was purchased from Coleção de Culturas Tropicais (Fundação André Tosello, Campinas, Brazil; synonym IFO10076). The yeast was reactivated and stored as described previously (Lopes et al., 2020a). The genomic DNA was extracted according to Otero et al. (2010). The DNA sequencing libraries were prepared with Illumina TruSeq Nano DNA Library Prep kit according to the manufacturer's recommendations. The libraries were sequenced on MiSeq v3 flow cell, read length 2 × 175 bp using the MiSeq platform (Illumina Inc., San Diego, CA, USA). The library preparation and sequencing were performed in the Institute of Genomics Core Facility, University of Tartu. Reads were cleaned and trimmed with Trimmomatic-0.39 (Bolger et al., 2014). Draft assembly was created with spades-3.12.0 (Bankevich et al., 2012) using diploid mode. Protein coding sequences were determined from received haplocontigs by homology search with blastn (Altschul, 1997) against reference NP11 gene sequences with coverage and identity thresholds 80% and 70%, respectively. This Whole Genome Shotgun project has been deposited at DDBJ/ENA/GenBank under the accession JABGON000000000. The version described in this paper is version JABGON010000000. For creating the native library parts and yeast transformations, the strain *R. toruloides* CCT7815 was used. This strain is derived from *R. toruloides* CCT0783 after a short-term adaptation in sugarcane bagasse (Bonturi et al., 2017a).

### 2.2 Plasmids

The backbone plasmid used for storing level 1 parts was either pSB1K3-RFP from the iGEM collection (http://parts.igem.org/Collections) or TOPO™ Vector (Thermo Fisher Scientific, Carlsbad, USA). For level 2 and level 3 plasmids, pSB1C3-RFP from the iGEM collection. When the GGA reactions using iGEM collection plasmids did not work, pGGA (New England Biolabs, New England Biolabs, Ipswich) containing a red fluorescent protein (RFP, BBa_E1010, http://parts.igem.org/Part:bba_E1010) (Celińska et al., 2017) was used instead. pSB1K3-RFP and TOPO vector carry resistance to the kanamycin gene, while pGGA and pSB1C3-RFP carry chloramphenicol resistance gene.

### 2.3 DNA sequences

The primer sequences used for removing internal BsaI cutting sites as well as to amplify all parts with their respective overhangs for the GGA can be found in Supplementary Table 1. The genomic DNA from *R. toruloides* was extracted (Lõoke et al., 2011) and used as a template for the amplification of insertional regions, promoters, terminators, and genes. For the insertional region, five hundred base pairs fragment upstream (insUP) and downstream (insD) the *KU70* (DNA-dependent ATP-dependent helicase subunit 70, RHTO_06014) were amplified from the genome. The promoters from the genes glyceraldehyde-3-phosphate dehydrogenase (*GAPDH*, also widely named as GPD1 in *R. toruloides*'s literature, RHTO_03746), alcohol dehydrogenase 2 (*ADH2*, RHTO_03062), xylose reductase (*XYL1*, RHTO_03963), glucose 6-phosphate isomerase (*PGI*, RHTO_04058), and 1,6-bisphosphatealdolase (*FBA*, RHTO_03043) (Díaz et al., 2018; Wang et al., 2016). The intronic promoter from the gene *LDP1* (lipid droplet protein 1 gene) (Liu et al., 2016) was synthesized (Twist Biosciences, San Francisco, USA) without BsaI recognition sites. The genes geranylgeranyl diphosphate synthase (*crtE*, RHTO_02504), phytoene dehydrogenase (*crtI*, RHTO_04602), and phytoene synthase/lycopene cyclase (*crtYB*, RHTO_04605) were amplified and used for proof of concept aiming at improving carotenoids production. BsaI recognition sites from the aforementioned genes were removed by inserting point mutations without changing the amino acids by overlapping PCR. For terminators, the sequences from cauliflower mosaic virus 35S (t35S, GenBank No. MF116009) and the *Agrobacterium tumefaciens* nopaline synthase (tNOS, MF116010) (Fillet et al., 2017) were synthesized (Twist Biosciences). Native terminators from heat shock 70 kDa protein 1/8 (tHSP, RHTO_07842) and the *GPD1* (tGPD) were amplified from the genomic DNA. The geneticin (*G418*), hygromycin (*HYG*), nourseothricin (*NAT*), and bleomycin (*BLE*) resistance genes were codon-optimized for *R. toruloides* using Benchling and synthesized either by IDT (Leuven, Belgium) or Twist Biosciences. When GC content was too high for synthesis and it was not possible to lower it without drastically changing the codons, the sequences were split into fragments for synthesis and assembled by either GGA or by overlapping PCR. The resistance genes were further assembled by GGA with *XYL1* promoter (pXYL1) and tNOS, herein called marker (M). All parts were submitted to Addgene.

### 2.4 DNA amplification by polymerase chain reaction (PCR)

DNA sequences below 3 kb were amplified via PCR using DreamTaq Green PCR Master Mix (2X) (Thermo Fisher Scientific, Vilnius, Lithuania) or HOT FIREPol GC Master Mix (Solis BioDyne, Tartu, Estonia), otherwise, the high-fidelities Phusion DNA Polymerase or Platinum SuperFi Master Mix (Thermo Fisher Scientific, Vilnius, Lithuania) were used instead. All reactions were set up and performed according to the manufacturer's instructions for a high GC content template. Following PCR, the samples were loaded on a 1% agarose gel and the electrophoresis at 120 V. The fragments sizes were estimated using gene ruler 1 kb Plus DNA Ladder (Thermo Fisher Scientific). Amplicons with the correct size were excised from the gel and purified using Favorprep™ GEL/PCR Purification Kit (Favorgen, Wien, Austria) following the manufacturer's instructions, quantified by Nanodrop (Thermo Fisher, Whaltan, USA), and used for GGA assembly and TOPO subcloning.

### 2.5 Construction of pGGA carrying a red fluorescence protein (RFP)

To facilitate the screening of bacterial colonies containing GGA constructs, a variant of pGGA containing the RFP flanked by overhangs and BsaI cutting sites was constructed. Both pGGA and RFP were amplified by PCR, purified, and assembled by Gibson Assembly (Gibson et al., 2009) reaction according to the manufacturer's instructions (Gibson Assembly Master Mix, New England Biolabs, Ipswich, USA). Two microliters of the assembly reaction were taken for the consequent bacterial transformation.

### 2.6 Assembly of levels 1, 2, and 3 constructs by GGA

**Fig. 1:**
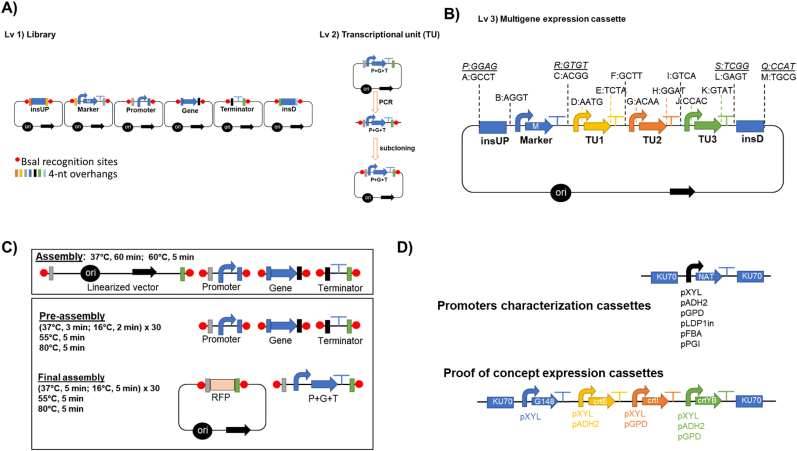
The RtGGA with **A)** Level 1 (Lv1) for the library of standardized parts-containing regions upstream (insUP) and downstream (insD) an insertional site, selection marker (M), promoters, genes, and terminators-and level 2 (Lv 2) by combining promoter (P), gene (G), terminator (T) into transcriptional units (TU). **B)** Level 3 (Lv3) RtGGA multigene expression cassette and standardized overhangs from the *Y. lipolytica* GGA platform (Celińska et al., 2017). The overhangs in *italic* are the changes made to make it compatible with *R. toruloides***C)** Upper box shows the reaction method proposed in the *Y. lipolytica* GGA platform (Celińska et al., 2017) for level 2 assembly using linearized PCR-amplified parts. The lower box shows the two-step assembly protocol adapted from Larroude et al., 2019 for the assembly of Lv2. **D)** Scheme of the cassettes assembled for the characterization of the promoters strength and the overexpression of the carotenoid pathway. Legend: ori – the origin of replication; black arrow– bacterial resistance gene; RFP: red fluorescence protein. (For interpretation of the references to colour in this figure legend, the reader is referred to the Web version of this article.)

Level 1 (Lv1, Fig. 1A) comprised the storage of single standardized parts (promoters, genes, markers, insertional regions, and terminators) in a plasmid. The PCR amplified part containing the overhang and flanking BsaI recognition sites were cloned to TOPO™ XL-2 Complete PCR Cloning Kit (Thermo Fisher Scientific, Vilnius, Lithuania) as shown in Fig. 1A.

The level 2 (Lv2, Fig. 1A) of GGA was the assembly of a gene (G) with a promoter (P) and terminator (T), resulting in a transcriptional unit (TU). For the proof of concept (Fig. 1B and D). of the GGA platform, the genes *crtE* (G1), *crtI* (G2), and *crtYB* (G3) were combined with three different promoters (pXYL1, pGPD1, and pADH2), and tNOS using the pSB1C3 or pGGA plasmid as vectors. A total of nine TUs were generated. The reactions were done using 150 ng of each part of the TU and either using: (i) 75 ng of vector, 2 μl of T4 DNA Ligase Buffer (New England Biolabs), 1 μl of T4 or T7 DNA Ligase (New England Biolabs), 1 μl of BsaI-HFV2 (New England Biolabs), up to 20 μl of nuclease-free H_2_O and the mixture was incubated at 37 °C for 1 h, followed by 5 min at 60 °C; or (ii) the NEB Golden Gate Assembly Mix (New England Biolabs) according to the manufacturer's instruction; or (iii) a two-step assembly protocol adapted from Larroude et al., 2019 using parts as amplicons (Fig. 1B) or into vectors.

The final step of the GGA platform is to assemble the pathway with a selection marker and the insertional regions for guiding the pathway integration with simultaneous deletion of a target gene (Fig. 1C). This structure was used for assembling both the cassette for the characterization of the strength of promoters from the library (pXYL1, pGPD1, pFBA, pADH2, pLDP1, and pPGI) and the overexpression of the carotenoid pathway (RtGGA proof of concept). The promoter strength was evaluated in terms of the expression of *NAT* gene as done similarly by Wang et al. (2016). Lv1 parts of *KU70* insUP and insD, *NAT*, tNOS, and promoters were assembled (Fig. 1D) by RtGGA in pGGA by reaction conditions described for library assembly.

For the proof of concept, *G418* marker was combined with the 3 TUs flanked by the insertional region aiming at the deletion of *KU70* gene (Fig. 1B and D). Three different level 3 constructs were obtained by using different combinations of promoters (Table 1). This was done by PCR amplification of Lv1 and Lv2 the aforementioned parts followed by subcloning them into PCR-XL-2-TOPO™ Vector (Thermo Fisher Scientific). The reaction for TOPO™ Cloning was modified as follows: 1 μl of PCR-XL-2-TOPO™ Vector, insert required for 5:1 M ratio of insert:vector, 1 μl of Salt Solution, up to 6 μl of deionized H_2_O. The mixture was incubated at 25 °C for 1 h. Two microliters of the cloning reaction were used for bacterial transformation. The reaction to assemble level 3 (Lv 3) GGA was prepared as follows: 75 ng of pGGA, the mass of each insert (TOPO™ plasmid) required for 2:1 M ratio of insert:vector, 2 μl of T4 DNA Ligase Buffer (New England Biolabs), 1 μl of T4 or T7 DNA Ligase (New England Biolabs, Ipswich, USA), 1 μl of BsaI-HFV2 (New England Biolabs), up to 20 μl of nuclease-free H_2_O. The reaction was performed as instructed by the official NEB GGA kit (37 °C for 5 min followed by 16 °C for 5 min) x 30 and 60 °C for 5 min.

**Table 1:** Yeast strains created by the integration of the different constructs done with RtGGA in *R. toruloides* CCT7815.

Strain number	Description
SBY92	Δ*ku70*:: pXYL-*G418*-tNOS-pXYL-*crtE*-tNOS-pGPD-*crtI*-tNOS-pADH2-*crtYB*-tNOS
SBY93	Δ*ku70*:: pXYL-*G418*-tNOS-pADH2-*crtE*-tNOS-pGPD-*crtI*-tNOS-pXYL-*crtYB*-tNOS
SBY94	Δ*ku70*:: pXYL-*G418*-tNOS-pADH2-*crtE*-tNOS-pXYL-*crtI*-tNOS-pGPD-*crtYB*-tNOS
SBY109	Δ*ku70*::pADH2-*NAT*-tNOS
SBY116	Δ*ku70*::pLDP1in-*NAT*-tNOS
SBY117	Δ*ku70*::pXYL-*NAT*-tNOS
SBY119	Δ*ku70*:: pGPD-*NAT*-tNOS
SBY124	Δ*ku70*:: pPGI-*NAT*-tNOS

All assemblies from GGA and TOPO™ subcloning were used for bacterial transformation using *Escherichia coli* DH5α according to the manufacturer's instructions. White colonies were screened by PCR using SP6/T7 and M13F/M13R primers (Supplementary Table 1), respectively. Colonies bearing constructs with the correct size were grown overnight using 4 ml of LB medium (10 g/l tryptone, 5 g/l yeast extract, 10 g/l NaCl) and the selection antibiotics. Plasmids containing the assemblies were recovered using Favorprep™ Plasmid DNA Extraction Mini Kit (Favorgen, Wien, Austria). DNA Sanger sequencing was used to confirm parts and constructs.

### 2.7 Yeast transformation and screening of correct transformants

*R. toruloides* was transformed according to the protocol described by Nora et al. (2019) with either the different level 3 constructs for the proof of concept or promoter strength characterization. *R. toruloides* CCT7815 (parental strain) was inoculated in 10 ml of YPD medium (glucose, 20 g/l; yeast extract, 10 g/l; peptone, 20 g/l) and incubated overnight with a stirring speed of 160 rpm at 30 °C. The culture was diluted to an OD600 of ∼0.2 in 10 ml of YPD and incubated again until an OD600 of ∼0.8. The culture was harvested in a sterile 50 ml centrifuge tube at 3000 *g* for 10 min. The cell pellet was resuspended in 25 ml of sterile water and centrifuged again. The cells were resuspended in 1 mL 100 mM lithium acetate (LiAc) (pH 7.5) and 500 μl of the suspension was transferred to a 1.5 ml microfuge tube. The cells were centrifuged at 3000 *g* for 30 s and the LiAc was removed. In the following order, 240 μl of polyethylene glycol (PEG) 4000 (50% w/v), 36 μl of 1M LiAc (pH 7.5), 24 μl of sterile water were added. Ten μl of salmon sperm DNA (10 mg/ml, pre-boiled at 100 °C for 10 min) was transferred to the tube before adding 50 μl of transforming DNA (0.1–10 μg, removed from the bacterial backbone by either restriction enzymes or by PCR amplification). The cell pellet and the added reagents were mixed completely by vigorous vortexing. After incubating the tube at 30 °C for 30 min, 34 μl of dimethyl sulfoxide (DMSO) was added to the mixture, which was then heat-shocked at 42 °C for 15 min. The tube was centrifuged at 3000 *g* for 30 s and the supernatant was removed. The pellet was resuspended in 2 ml of YPD and transferred to a new 15 ml tube, which was incubated overnight with shaking at 30 °C. The following day the suspension was plated onto solid YPD containing selection pressure and left to grow at 30 °C for two days. For the strains transformed with level 3 carotenoids multigene expression cassette G418 was used, while for the promoter characterization *NAT* was used (100 mg/ml for pPGI and pXYL1, 50 mg/ml pADH2 and pFBA, 25 mg/ml pLDP1 and pGPD1).

For the screening of correct transformants, yeast colonies were checked by PCR and sequencing. The yeast colony was grown overnight in 10 ml of YPD and the gDNA was extracted using the Wizard Genomic DNA Isolation kit (Promega, Switzerland). The PCR reaction was as follows: 40 ng of template, 5 μl Platinum SuperFi II Master mix (Thermo Fisher Scientific, Vilnius, Lithuania), 2 μl of SuperFi GC enhancer (Thermo Fisher Scientific, Vilnius, Lithuania), 1 μmol/l each of reverse and forward verification primers (Supplementary Table S1), and water to 10 μl. Reaction conditions were done according to the manufacturer's instructions. The amplicons were evaluated by electrophoresis and the ones with the correct size were purified from the agarose gel and sequenced.

### 2.8 Characterization of the strains in shake flasks

Xylose was used as a carbon source for the characterization of strains presented in Table 1. Mineral medium, containing, per liter, 30.0 g carbon source, 0.8 g (NH_4_)_2_SO_4_, 3.0 g KH_2_PO_4_, 0.5 g MgSO_4_·7 H_2_O, 1.0 ml vitamins solution, and 1.0 ml trace metal solutions were used (Lahtvee et al., 2017). The carbon to nitrogen (C/N) ratio of the medium was 80 (mol/mol). The cells were incubated at 30 °C and at a stirring speed of 160 rpm until xylose depletion. For all the strains, samples were taken for the yeast characterization in terms of growth (absorbance at 600 nm; OD600) and RNA extraction (mid-exponential phase). Strains *R. toruloides* CCT7815 and SBY92, 93 and 94 were also characterized in terms of metabolites profile (HPLC) and production of carotenoids.

### 2.9 RNA extraction and cDNA synthesis

Materials used were RNase free and all steps were done at room temperature unless stated otherwise. One milliliter aliquots of the cell liquid culture from the mid-exponential phase (OD600 between 14 and 18) was collected and harvested at 23,000 *g* for 30 s at 4 °C. After centrifugation supernatant was quickly discarded and the cell pellet was frozen in liquid nitrogen. Samples were stored at −80 °C. RNA extraction was done using the Tri reagent kit (Invitrogen, USA). A few modifications were done to the protocol that was provided by the manufacturer. Before cell lysis in 1 ml of Tri reagent, the cell pellet was washed twice in 1 ml of RNAse free water using centrifugation as described above. One milliliter of Tri reagent was added to the biomass, which was resuspended by pipetting and incubated for 5 min followed by the addition of 200 μl of chloroform. The suspension was thoroughly mixed using a vortex, followed by 15 min of incubation. A triphasic system was obtained after centrifugation at 12,000 *g* for 15 min at 4 °C. The upper aqueous phase containing the RNA was transferred to a new tube and 0.5 ml of ice-cold 2-propanol was added. The mixture was incubated for 10 min and the RNA pellet was recovered after centrifugation at 12,000 *g* for 15 min at 4 °C and washed with 1 mL of ice-cold 75% (v/v) ethanol. After removing the supernatant, the RNA pellet was dried and 50 μL of RNA-free water was added for resuspension. RNA was kept at −80 °C until the cDNA synthesis which was done using the First Strand cDNA Synthesis Kit (Thermo Scientific, USA) according to the manufacturer's instructions for GC-rich templates. Five hundred nanograms of RNA were used for the cDNA synthesis.

### 2.10 Measurement of relative expression of promoters and carotenogenic genes by real-time PCR (RT-PCR)

Relative gene expression by RT-PCR was used to determine the strength of six native promoters and to validate the overexpression of the carotenogenic pathway. Promoter strength was measured by the expression of *NAT* gene and pADH2 was chosen as reference. For the assessment of the overexpression of native *crtE*, *crtI*, and *crtYB*, *R. toruloides* CCT7815 (parental strain) was chosen as a reference. *GAPDH* (glyceraldehyde 3-phosphate dehydrogenase) was used as an endogenous control (Bonturi et al., 2017a, Bonturi et al., 2017b). Three biological and five technical replicates were used. The resulting cDNA from section 2.9 was diluted 2.5 times right before use. The reaction was done as follows: 2 μl of HOT FIREPol® EvaGreen® qPCR Supermix (EG) (SolisBiodyne, Estonia), 3 mmol/l of each forward and reverse primer (Supplementary Tables 1) and 1 μl cDNA and Milli Q purified water to a final volume of 10 μl. Reaction conditions were: 95 °C for 12 min and 45 x (95 °C for 15 s, 64 °C for the 20 s, 72 °C for 20 s). The gene relative gene expression was calculated as described in Bonturi et al., 2017a, Bonturi et al., 2017b.

### 2.11 Analytical methods

Microbial growth was estimated by measuring OD600 nm and converted to biomass concentration by using a calibration curve. Concentrations of metabolites, such as xylose, organic acids and glycerol were measured using high-pressure liquid chromatography (HPLC) (LC-2050C, Shimadzu, Kyoto, Japan) equipped with HPX-87H column (Bio-Rad, CA, USA) and a refractive index detector (RID-20A, Shimadzu, Kyoto, Japan), at 45 °C and 5 mmol/l H_2_SO_4_ as mobile phase with isocratic elution at 0.6 ml/min.

Once xylose was depleted completely the final cell biomass was recovered by centrifugation at 4000 *g* for 10 min and washed twice with distilled water. Cells were frozen at −80 °C overnight and lyophilised. The final dry cell mass was determined gravimetrically.

Carotenoids were extracted from lyophilised biomass as described in Pinheiro et al. (2020). Quantification was done by measuring absorbance at 450 nm (Larroude et al., 2018) in a spectrophotometer and also by adapting to HPLC an ultra-performance liquid chromatography (UPLC) method described in Pinheiro et al. (2020). The separation was done at 40 °C in a C18 column (Kinetex 2.6 μm C18 100 Å, 100 × 4.6 mm, Phenomenex, Torrance, USA) using isocratic elution with acetone:water (70:30, % v/v) for 10 min , followed by a gradient to 100% of acetone in 2 min, and re-equilibration for 5 min using acetone:water (70:30, % v/v). A flow rate of 0.8 ml/min was used. The UV detector was set to 40 °C and 450 nm. Detected peaks were identified according to the retention time profile determined by Weber et al. (2007). β-carotene (Alfa Aesar, MA, USA) was used for the construction of the calibration curve for both methods.

## 3 Results and discussion

### 3.1 Genome sequence of *R. toruloides* CCT 0783

The genome of *R. toruloides* CCT0783 was sequenced in order to identify BsaI recognition sites and to allow the design of primers for the amplification of insertional regions, promoters, terminators and genes. llumina MiSeq platform was used, and the sequence was deposited (https://www.ncbi.nlm.nih.gov/genome/13280?genome_assembly_id=1549609). The total GC content was as expectedly high (61.9%) while the length of 40.59 Mb was determined. The determined length is about double the size of the genome of other *R. toruloides* strains deposited in NCBI (about 20 Mb; https://www.ncbi.nlm.nih.gov/genome/browse#!/eukaryotes/13280/). *R. toruloides* CGMCC 2.1609 was the only strain with an intermediate genome size of 33.39 Mb. About 55% of the genome of this strain posses ≥99% identity to the genome of haploid strain CBS 14 (A1 mating-type; synonym to IFO 0559) while 41.6% is highly similar to the strain IFO 0880 (A2 mating-type; synonym to CBS 349) (Sambles et al., 2017b). A similar finding was obtained with the genome of *R. toruloides* CCT 0783, where two versions of the same gene were found, one presenting ≥90% identity and the other version presenting ≥70% identity to the genome of haploid strain NP11. Further studies and analysis are being carried out to understand the ploidy, phylogenetic tree, and which of the versions of the genes are being expressed. Since the goal of this work was to develop a standardized, robust, and versatile platform for golden gate assembly for *R. toruloides* (RtGGA), the genomic DNA of the strain used in this work was used for designing primers, accessing the presence of BsaI recognition sites, and validating the constructs by Sanger sequencing. For parts amplification, the genomic DNA from *R. toruloides* CCT7815 was used instead, as this strain is derived from CCT0783 and has higher traits in lignocellulosic hydrolysates.

### 3.2 Constructing standardized biological parts for RtGGA

The foundation of synthetic biology lies in standardized biological parts that would allow us to build new biological systems more efficiently and offer better designs for metabolic engineering approaches. The YTK for *S. cerevisiae*, a diverse collection of parts, exemplifies how crucial is standardization for synthetic biology. The YTK has a wide range of regulatory elements and selection markers, which makes engineering the genome and fine-tuning gene expression much less laborious. Expanding this technology to a wider range of microorganisms would allow swift metabolic engineering for wider biotechnology applications. Maintaining similar standards for assemblies is also helpful for exchanging parts between different yeasts.

With this goal in mind, we have developed a dedicated GGA platform for *R. toruloides*, inspired by the YTK standardized overhangs and the multilevel assembly structure and various parts collections developed for other yeasts (Celińska et al., 2017; Prielhofer et al., 2017; Rajkumar et al., 2019). Supplementary Tables 2 and 3 summarizes and compares different yeast GGA-dedicated platforms. Due to large differences in the genus of *R. toruloides* and other yeasts with dedicated GGA platforms, it was necessary to extensively optimize almost all steps of the assembly. *R. toruloides* has a GC-rich genome, leading to a plethora of problems, from extremely high melting temperatures during primer design to the formation of secondary structures. As *R. toruloides* parts used in this work had several cutting sites from different Type IIS enzyme, the strategy of blunt-subcloning amplicons containing overhangs and BsaI cutting sites was preferred over removing extra cutting sites by overlapping PCR or synthesizing the genes. Using parts subcloned into plasmids was shown to be more efficient for GGA than when using linearized parts, as the vector backbone allowed the restriction enzyme to anchor to the DNA more efficiently. Even the TOPO™ Cloning technology that had been developed for easy cloning, it was required to optimize the established method to accommodate the GC-rich genome of *R. toruloides*.

A dedicated GGA platform enables fast construction of complex expression cassettes for metabolic engineering in *R. toruloides*. A set of thirteen 4-nucleotide (nt) overhangs from the *Y. lipolytica* GGA system (Celińska et al., 2017) was initially used for this task. This set covered all three transcription units (TUs, containing P – promoter, G – gene, T - terminator), selection marker M (also a TU with P-G-T), and upstream and downstream insertional units, insUP and insD, respectively (Fig. 1D).

For the assembly of level 1, the parts and the vector pSBIK3_RFP from the iGEM collection were used. For each position, the plasmid, along with the sequence to be used as the building block of the GGA, was PCR amplified using the corresponding primers containing the BsaI recognition site and the 4-nt overhang (Supplementary Table 1). In the case of a successful round of GGA reaction and transformation, the original red colonies of the pSBIK3_RFP plasmid were changed to white ones, as the GGA building block was inserted instead of the red fluorescence protein (RFP) reporter gene, therefore, allowing a quick and easy pre-screening.

The parts library (Fig. 1A) was successfully assembled and contained: (i) six promoters (pGPD1, pXYL1, pADH2, pLDP1in, pPGI, pFBA); (ii) three native genes from the carotenogenic pathway (*crtE*, *crtI*, and *crtYB*); (iii) terminators (tNOS, t35S, tHS, tGPD); (iv) insertional regions, 500 bp upstream and downstream the *KU70* gene, insUP and insD, respectively; and (v) resistance genes (*G418*, *NAT*, *BLE*, *HYG*). All parts were verified by DNA sequencing.

As for the Level 2, nine TUs were assembled by having all three carotenogenic genes under the three different promoters (Fig. 1A). The TUs were assembled by using diverse approaches: (i) either using the official GGA kit or by using enzymes separately (BsaI and T4 or T7 ligase); (ii) different plasmids (pSB1C3_RFP or pGGA_RFP); and (iii) either by one or two-step protocol (Larroude et al., 2019).

The RtGGA found to be a robust system regardless of the assembly strategy, backbone, and the usage of the official kit or not, but with the increasing assembly complexity and high-GC content DNA, a higher efficiency was obtained by using a two-step assembly protocol (Fig. 1D). The two-step assembly method most likely avoids unspecific ligations between overhangs. All nine TUs were assembled effectively and confirmed by sequencing. Once the level 2 construction was successful, the cassette for promoter strength characterization (Fig. 1D) was built and transformed in the parental strain (*R. toruloides* CCT7815). This assessment would further be used for selecting the promoters for assembling the multigene cassette.

### 3.3 Characterizing promoters for *R. toruloides*

**Fig. 2:**
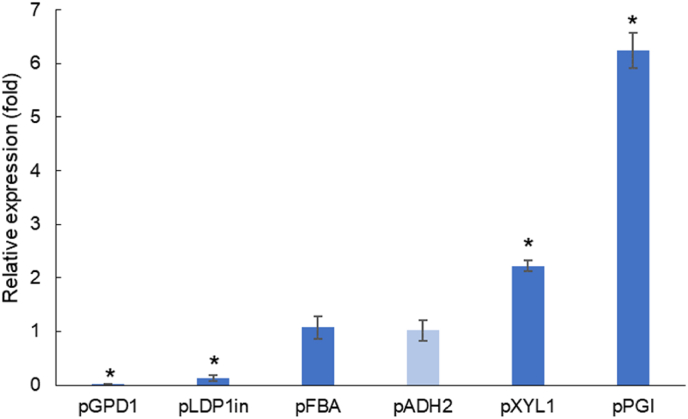
Relative expression in terms of the fold of the NAT gene under different promoters. pADH2 (light blue) was used as a reference and the asterisk indicates a difference with statistical significance (Student's *t*-test; p-value ≤ 0.05). (For interpretation of the references to colour in this figure legend, the reader is referred to the Web version of this article.)

As the *R. toruloides* CCT 0783 strain displayed differences in terms of its genome size, and probably ploidy or in terms of expression of genes as two different versions of the same gene were identified, we decided to characterize the promoter strength in its derived strains. Six native promoters were selected based on previous works with *R. toruloides* (Liu et al., 2016; Wang et al., 2016; Díaz et al., 2018; Nora et al., 2019), assembled with *NAT* gene, tNOS and flanking regions (insUP and insD) targeting the KU70 loci for integration. The six different cassettes were transformed into *R toruloides* and the resulting strains were grown in minimal medium and xylose as carbon source. Biomass samples were collected from the exponential growth phase for the promoters strength characterization in terms of relative expression of *NAT* gene by using RT-PCR. The promoter ADH2 was chosen as a control as its *NAT* expression had the lowest standard deviation among the biological replicates. The order of strength in xylose-grown exponential cells was pGPD < pLDP1in < pADH2 = pFBA < pXYL < pPGI (Fig. 2). Díaz et al. (2018) also reported pXYL as being a stronger promoter than pGPD for *R. toruloides* CECT 13085 for xylose-cultivated cells regardless of the growth phase. For *R. toruloides* AS 2.1389 grew on YPD medium the promoter strength order was pGPD < pFBA < pPGI (Wang et al., 2016). Liu et al. (2016) reported stronger activity of pLDP1in when compared to pGDP1 in glucose-grown *R. toruloides* ATCC 10657. Nora et al. (2019) characterized a wide range of native promoters for *R. toruloides* IFO0880 and pGPD1 was defined to be a medium-strong promoter. The authors also reported that pGDP1 was shown to have less predictable expression levels than other promoters accessed, as the levels were different depending on the reporter gene and cultivation medium. Differences in promoter strength found in different literature might be explained by using different strains, media, and characterization methodologies, as some works measured expression by the fluorescence produced by expressing reporter proteins (Nora et al., 2019; Díaz et al., 2018; Liu et al., 2016) or by a reporter gene relative expression using RT-PCR (Wang et al., 2016). A weak (pGPD1), a medium (pADH2), and a strong (pXYL1) promoter were selected to be tested further in the proof of concept cassette.

### 3.4 Proof of concept of RtGGA for the overproduction of carotenoids

At first, for level 3 construction of the proof of concept cassette, it was required to assemble into GGA plasmid the following PCR amplified parts: insUP, Marker, TU1, TU2, TU3, and insD. The initial assembling of the level 3 construct turned out to be unsuccessful using GGA plasmid, while different concentrations of enzyme (BsaI and T7 or T4 DNA ligase) and buffer, different pre-assembly strategies, or even switching to the official NEB GGA kit were tested. To overcome the issue, new overhangs (R and S) were designed with no potential similarity with each other. As recommendations, it is suggested to simulate *in silico* GGA to check for possible problems regarding the overhangs and the assembly. For the Lv2, after the TU was assembled by GGA, the new constructs were PCR amplified to re-insert BsaI flanking recognition sites and subcloned to TOPO vectors (Fig. 1B). While, for the subcloning of level 1 parts (insUP and insDO) worked by using the TOPO™’s manufacturer's protocol, but for subcloning the TUs and marker it was required to optimize the protocol. Different ratios of TU fragment to TOPO™ vector were used, and a 5:1 M ratio was found to be the most efficient (data not shown). Three different level 3 constructs containing the *crtE*, *crtI*, and *crtYB* genes under different combinations of pXYL, pGPD, and pADH2, respectively, were assembled by GGA by the one-step reaction. Using parts inside plasmids probably increases the efficiency of BsaI recognition and cutting by offering a higher surface for the enzyme to dock on. In case of failure of the GGA by using the manufacturer's protocol, it is suggested to use the two-step or other types of pre-assemblies.

**Fig. 3:**
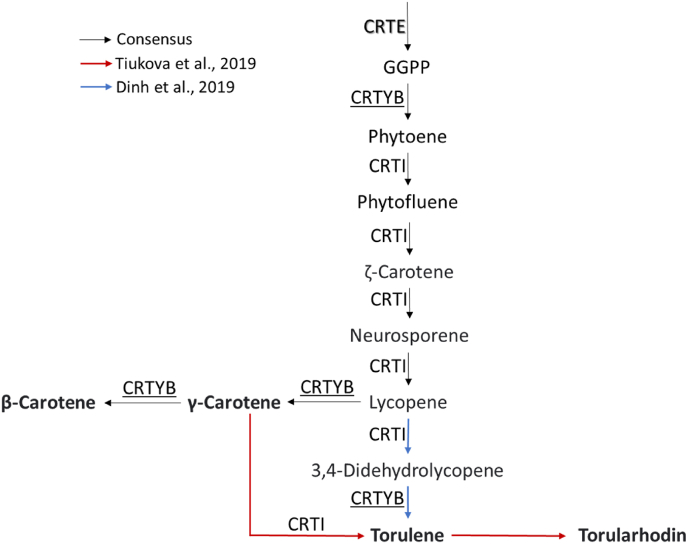
Carotenoid biosynthesis in *R. toruloides* according to different genome-scale models (GEM) (Dinh et al., 2019; Kim et al., 2021; Tiukova et al., 2019). Geranylgeranyl diphosphate synthase (CRTE) catalyzes the formation of precursor of carotenoids. Phytoene dehydrogenase (CRTI) and phytoene synthase/lycopene cyclase (CRTYB) are both involved in the production of, respectively, acyclic and cyclic carotenoids.

The efficiency of the developed GGA was validated by overexpressing the three most important genes in the carotenoids production pathway - *crtE*, which catalyzes the formation of an important precursor of carotenoids; *crtI*, and *crtYB*, both of which are involved in the production of γ-carotene, β-carotene, torularhodin and torulene (Fig. 3). In this work, we aimed to validate the RtGGA by regulating the expression of these genes under different combinations of promoters, such as the strong xylose reductase (pXYL1), mild alcohol dehydrogenase 2 (pADH2), and weak glyceraldehyde 3-phosphate dehydrogenase (pGPD1). The constructions were transformed into *R. toruloides* CCT7815 and three new strains were obtained: SBY92, SBY93, and SBY94 (Table 1 and Fig. 1D).

**Fig. 4:**
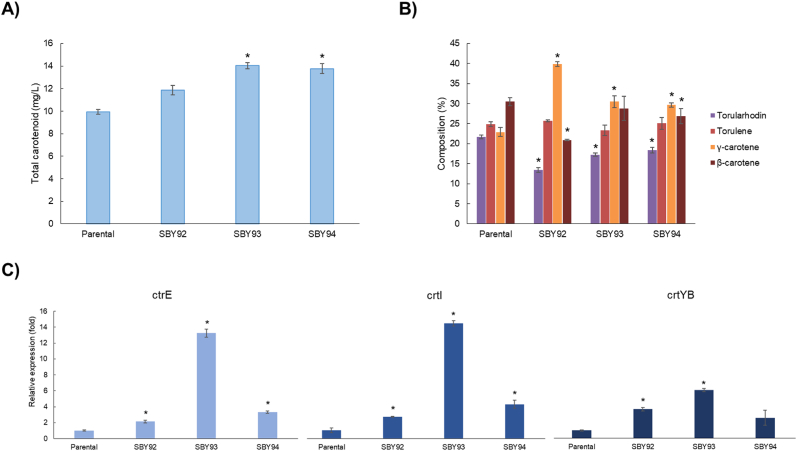
**A)** final carotenoid titer and **B)** composition (HPLC method). **C)** Relative expression of the genes crtE, crtI, and crtYB in the strains SBY92-94 in comparison to parental strain during the exponential phase. Asterisk shows the significative statistical difference (Student's *t*-test; p-value ≤ 0.05) when compared to the parental strain (*R. toruloides* CCT 7815).

When compared to the parental strain (*R. toruloides* CCT7815), only SBY92 presented differences in biomass production (Supplementary Fig. S1). The xylose consumption profile (Supplementary Fig. S1) and the maximum specific growth rates (μ_max_) were identical for all strains (*R. toruloides* CCT7815, and SBY93: 0.12 ± 0.00 1/h and SBY94: 0.12 ± 0.01 1/h), except for SBY92 which was statistically significant slightly lower (μ_max_: 0.11 ± 0.00 1/h). According to the quantification done with the HPLC method (same used for previous publications on *R. toruloides* CCT7815), the parental strain produced 9.9 ± 0.2 mg/l, while all three modified strains produced 11.9 ± 1.3, 14.0 ± 0.3, 13.8 ± 0.5 mg/l of total carotenoids, respectively (Fig. 4A). Concentrations measurements using the spectrophotometer were on average 41% higher than the ones using the HPLC method, probably due to the presence of other intermediate carotenoids that were not detected with the latter.

SBY92 was the only modified strain that displayed a statistically significant improvement in carotenoid yield on biomass (Y_X/CAR_: 2.01 ± 0.06 mg carotenoid/g dry cell mass) when compared to the parental strain (Y_X/CAR_: 1.23 ± 0.04 mg carotenoid/g dry cell mass), probably due to the lower achieved biomass (Supplementary Fig. S1). Regarding carotenoid composition (Fig. 4B), all modified strains presented lower torularhodin and increased γ-carotene percentages in the total carotenoid composition than the parental strain. SBY94 was found to have an increased β-carotene percentage. SBY92-94 also displayed higher expression of *crt*E, *crt*I, and *crt*YB, validating the overexpression of the pathway genes (Fig. 4C).

A Spearman correlation (ρ) was run to assess the monotonic relation between the expression of *crt*E, *crt*I, and *crt*YB and carotenoid titers. The correlations were very strong for *crtE* and *crtI* (ρ; *crtE*: 0.88; *crtI*: 0.93; p-value; *crtE*: 0.004; *crtI*: 0.001; n: 8) and strong for *crtB* (ρ: 0.76; p-value: 0.028; n: 8). The non-linearity of these correlations could be related to bottlenecks in the supply of geranylgeranyl diphosphate (GGPP) precursors (Fig. 3). In this case, an increase in the expression of the carotenogenic genes would be beneficial only until the available *crtE* and *crtI* are saturated with their substrates. After this point, there is an excess enzyme, but the amount of active enzyme remains the same, as does the maximum rate of reaction. Considering that SBY94 relative expressions of *crtE*, *crtI*, and *crtYB* are, respectively, 4.0, 3.4, and 2.4 lower than SBY93 (Fig. 4C), yet the carotenoid titer produced by these strains is statistically the same (Fig. 4A), we suggest that expression levels higher than those presented by SBY93 would no longer be beneficial without the overexpression of genes upstream of *crtE*. Genes from the mevalonate pathway, such as HMG-CoA reductase, are recommended targets for overexpression to increase carotenoid production (Tiukova et al., 2019) and have already shown positive results for this strategy for other carotenogenic yeats (Wang and Keasling, 2002). Besides that, the recent finding that the major bottleneck on beta-carotene production in *Y. lipolytica* was the substrate inhibition of the enzyme lycopene cyclase, even when using a heterologous pathway (Ma et al., 2022). The carotenogenic pathway in *R. toruloides* is not fully elucidated and probably is also subjected to inhibitions.

The relative expression of *crtE*, *crtI*, and *crtYB* genes may also be affecting the distribution between carotenoids derived from lycopene; γ-carotene and β-carotene, and 3,4-dihehydrolycopene; torulene and torularhodin (Fig. 3). This classification assumes that γ-carotene is not converted to torulene by crtI (Dinh et al., 2019; Hausmann and Sandmann, 2000; Schaub et al., 2012). SBY92 and SBY93 have accumulated proportionally more γ-carotene and β-carotene than parental strain and SBY94 (Table 2). Lycopene is both substrate and inhibitor of lycopene cyclase, present in *crtYB* (Ma et al., 2022). The inhibition increases and β-carotene selectivity decreases proportionally to the lycopene rate of formation (Ma et al., 2022), which is mediated by *crtI* (Fig. 3). Therefore, selectivity towards γ-carotene/β-carotene is proportional to the active *crtYB/crtI* ratio. Those were calculated for *R. toruloides* CCT7815 and SBY92-94 using their respective relative expression values (Table 2; Fig. 4C). For this calculation, we considered it reasonable to use SBY94's relative *crtI* expression also for SBY93's ratio, due to the reasons discussed in the previous paragraph. The calculation results were consistent with what was observed experimentally: SBY92 and SBY93 have higher crtYB/crtI ratios and γ-carotene/β-carotene selectivity.

**Table 2:** Carotenoid titer and expression gene ratios for *R. toruloides* CCT7815 and SBY92-94.

Strain	γ-car + β-car/trl + trlhd	*crtYB/crtI*
parental	1.15 ± 0.014^a^	1.03 ± 0.34
SBY92	1.55 ± 0.025^b^	1.34 ± 0.08
SBY93	1.46 ± 0.101^b^	1.43 ± 0.03^c^
SBY94	1.30 ± 0.100^a^	0.58 ± 0.21^b^

γ-car: γ-carotene; β-car: β-carotene; trl: torulene; trlhd: torularhodin.

a Statistically the same.

b Statistically the same.

c Calculated with crtI ratio of SBY94.

The concentrations for carotenoids produced by *R. toruloides* grown on glucose range from 14.0 to 33.4 mg/l (Dias et al., 2015). *R. toruloides* CCT7815 has been reported previously to produce 1.1 and 1.72 mg/l of total carotenoids in xylose-rich hydrolysates from sugarcane bagasse and birch, respectively (Bonturi et al., 2017b; Oliveira et al., 2021). In pure xylose cultivation in bioreactors, this strain was able to produce 20.7 and 36.2 mg/l without and under light irradiation (Pinheiro et al., 2020). For *R. glutinis* cultivated in brewery effluent, the literature reports 1.2 mg/l total carotenoids (Schneider et al., 2013). By optimizing the fermentation conditions, changing C/N ratios, and doing several rounds of metabolic engineering, *Y. lipolytica* was able to produce 33 mg/g dry cell mass of carotenoids (Gao et al., 2017). Using optimum promoter-gene pairs for heterologous carotenoid production pathway, Larroude et al. (2018) were able to engineer a strain of *Y. lipolytica* with a maximum yield of 90 mg/g dry cell mass of β-carotene. Literature uses either HPLC or spectrophotometric methods to quantify carotenoids but results are divergent. Liu et al. (2021) presented that, even when using the same methodology, different carotenoids as standards led up to 40% variation in the results. Therefore, it is suggested the development of a standard methodology to allow a fair comparison between different strains and studies from different groups. The titers of total carotenoids achieved in this work are superior or within the expected margin of concentrations attained in non-optimized strains and cultivation conditions. However, to transform *R. toruloides* into a competitive biotechnological producer of carotenoids, it is necessary to further fine-tune the expression of the carotenoids synthesis pathway, elimination of competing pathways, alleviation of substrate inhibition, and as well as optimize the cultivation conditions.

## 4 Conclusions

In this work, the existing collection of YTK for different yeasts was adapted to *R. toruloides*, requiring optimization of almost all steps of the assembly, which is, to our knowledge, the first dedicated GGA for a basidiomycete. The RtGGA platform was further validated by the assembly of a construct consisting of three genes, a selection marker, and insertional units. The three genes, coding for essential proteins in the carotenoids biosynthesis pathway were overexpressed and led to increased carotenoid production (up to 41%). Increased gene expression was confirmed by RT-PCR. The establishment of a golden gate assembly platform for *R. toruloides* fills a gap of advanced genome engineering tools for this non-conventional yeast that has engineering potential for industrial biotechnology applications. Optimization of cultivation conditions to increase carotenoid yield, as well as using the now established GGA platform to help with overexpressing key proteins in the lipid production pathway can make *R. toruloides* an industrial yeast capable of producing fine chemicals and biofuels, helping in the transition towards a sustainable economy.

## Author statement

NB, MJP, RLA, P-JL designed the experiments. NB, MJP, ER, PMO, GL, and TE performed the experiments. AB and MR performed the assembly and annotation of the genome. NB, MJP, PMO, GL, EAM, RLA, P-JL analyzed the data. NB, ER, JSDB, and MJP wrote the manuscript. All authors revised the manuscript.

## Funding

This project receives funding from the Bio-based Industries Joint Undertaking (JU) under the 10.13039/501100007601European Union’s Horizon 2020 research and innovation programme under grant agreement No 101022370. The JU receives support from the European Union's Horizon 2020 research and innovation programme and the Bio-based Industries Consortium. The project also received funding from 10.13039/501100007601European Union’s Horizon 2020 research and innovation program under grant agreement No 668997, the 10.13039/501100002301Estonian Research Council (grants PUT1488P and PRG1101), 10.13039/501100002322Coordination for the Improvement of Higher Education Personnel (Capes), São Paulo Research Foundation (FAPESP, grant 2016/10636-8), and DORA Plus. And support from 10.13039/501100000921COST Action CA-18229 “Yeast4bio”. AB and MR were funded by the EU ERDF grant No. 2014-2020.4.01.15-0012 (Estonian Centre of Excellence in Genomics and Translational Medicine).

## Declaration of competing interest

The authors declare that they have no known competing financial interests or personal relationships that could have appeared to influence the work reported in this paper.

## References

[bib1] Adrio J.L. (2017). Oleaginous yeasts: promising platforms for the production of oleochemicals and biofuels. Biotechnol. Bioeng..

[bib2] Altschul S. (1997). Gapped BLAST and PSI-BLAST: a new generation of protein database search programs. Nucleic Acids Res..

[bib3] Bankevich A., Nurk S., Antipov D., Gurevich A.A., Dvorkin M., Kulikov A.S., Lesin V.M., Nikolenko S.I., Pham S., Prjibelski A.D., Pyshkin A.V., Sirotkin A.V., Vyahhi N., Tesler G., Alekseyev M.A., Pevzner P.A. (2012). SPAdes: a new genome assembly algorithm and its applications to single-cell sequencing. J. Comput. Biol..

[bib4] Bolger A.M., Lohse M., Usadel B. (2014). Trimmomatic: a flexible trimmer for Illumina sequence data. Bioinformatics.

[bib5] Bonturi N., Crucello A., Viana A.J.C., Miranda E.A. (2017). Microbial oil production in sugarcane bagasse hemicellulosic hydrolysate without nutrient supplementation by a *Rhodosporidium toruloides* adapted strain. Process Biochem..

[bib6] Bonturi N., Crucello A., Viana A.J.C., Miranda E.A. (2017). Microbial oil production in sugarcane bagasse hemicellulosic hydrolysate without nutrient supplementation by a *Rhodosporidium toruloides* adapted strain. Process Biochem..

[bib7] Celińska E., Ledesma-Amaro R., Larroude M., Rossignol T., Pauthenier C., Nicaud J.M. (2017). Golden Gate Assembly system dedicated to complex pathway manipulation in *Yarrowia lipolytica*. Microb. Biotechnol..

[bib8] Dias C., Sousa S., Caldeira J., Reis A., Lopes da Silva T. (2015). New dual-stage pH control fed-batch cultivation strategy for the improvement of lipids and carotenoids production by the red yeast *Rhodosporidium toruloides* NCYC 921. Bioresour. Technol..

[bib9] Díaz T., Fillet S., Campoy S., Vázquez R., Viña J., Murillo J., Adrio J.L. (2018). Combining evolutionary and metabolic engineering in *Rhodosporidium toruloides* for lipid production with non-detoxified wheat straw hydrolysates. Appl. Microbiol. Biotechnol..

[bib10] Dinh H.v., Suthers P.F., Chan S.H.J., Shen Y., Xiao T., Deewan A., Jagtap S.S., Zhao H., Rao C.v., Rabinowitz J.D., Maranas C.D. (2019). A comprehensive genome-scale model for *Rhodosporidium toruloides* IFO0880 accounting for functional genomics and phenotypic data. Metabolic Engineering Communications.

[bib11] Engler C., Gruetzner R., Kandzia R., Marillonnet S. (2009). Golden gate shuffling: a one-pot DNA shuffling method based on type Ils restriction enzymes. PLoS One.

[bib12] Engler C., Kandzia R., Marillonnet S. (2008). A one pot, one step, precision cloning method with high throughput capability. PLoS One.

[bib13] Fillet S., Ronchel C., Callejo C., Fajardo M.J., Moralejo H., Adrio J.L. (2017). Engineering *Rhodosporidium toruloides* for the production of very long-chain monounsaturated fatty acid-rich oils. Appl. Microbiol. Biotechnol..

[bib14] Gao S., Tong Y., Zhu L., Ge M., Zhang Y., Chen D., Jiang Y., Yang S. (2017). Iterative integration of multiple-copy pathway genes in *Yarrowia lipolytica* for heterologous β-carotene production. Metab. Eng..

[bib15] Gibson D.G., Young L., Chuang R.-Y., Venter J.C., Hutchison C. a, Smith H.O., Iii C.A.H., America N. (2009). Enzymatic assembly of DNA molecules up to several hundred kilobases. Nat. Methods.

[bib16] Hausmann A., Sandmann G. (2000). A single five-step desaturase is involved in the carotenoid biosynthesis pathway to β-carotene and torulene in *Neurospora crassa*. Fungal Genet. Biol..

[bib17] Hu J., Ji L. (2016). Draft genome sequences of *Rhodosporidium toruloides* strains ATCC 10788 and ATCC 10657 with compatible mating types. Genome Announc..

[bib18] Johns A.M.B., Love J., Aves S.J. (2016). Four inducible promoters for controlled gene expression in the oleaginous yeast *Rhodotorula toruloides*. Front. Microbiol..

[bib19] Kim J., Coradetti S.T., Kim Y.M., Gao Y., Yaegashi J., Zucker J.D., Munoz N., Zink E.M., Burnum-Johnson K.E., Baker S.E., Simmons B.A., Skerker J.M., Gladden J.M., Magnuson J.K. (2021). Multi-omics driven metabolic network reconstruction and analysis of lignocellulosic carbon utilization in *Rhodosporidium toruloides*. Front. Bioeng. Biotechnol..

[bib20] Koh C.M., Liu Y., Moehninsi Du M., Ji L. (2014). Molecular characterization of KU70 and KU80 homologues and exploitation of a KU70-deficient mutant for improving gene deletion frequency in Rhodosporidium toruloides. BMC Microbiol..

[bib21] Kot A.M., Błazejak S., Gientka I., Kieliszek M., Bryś J. (2018). Torulene and torularhodin: “New” fungal carotenoids for industry?. Microb. Cell Factories.

[bib22] Koutinas A.A., Chatzifragkou A., Kopsahelis N., Papanikolaou S., Kookos I.K. (2014). Design and techno-economic evaluation of microbial oil production as a renewable resource for biodiesel and oleochemical production. Fuel.

[bib23] Kumar S., Kushwaha H., Bachhawat A.K., Raghava G.P.S., Ganesan K. (2012). Genome sequence of the oleaginous red yeast *Rhodosporidium toruloides* MTCC 457. Eukaryot. Cell.

[bib24] Lahtvee P.-J., Sánchez B.J., Smialowska A., Kasvandik S., Elsemman I.E., Gatto F., Nielsen J. (2017). Absolute quantification of protein and mRNA abundances demonstrate variability in gene-specific translation efficiency in yeast. Cell Syst..

[bib63] Larroude M., Celinska E., Back A., Thomas S., Nicaud J.M., Ledesma‐Amaro R. (2018). A synthetic biology approach to transform Yarrowia lipolytica into a competitive biotechnological producer of β‐carotene. Biotechnol. Bioeng..

[bib25] Larroude M., Park Y.K., Soudier P., Kubiak M., Nicaud J.M., Rossignol T. (2019). A modular Golden Gate toolkit for *Yarrowia lipolytica* synthetic biology. Microb. Biotechnol..

[bib26] Ledesma-Amaro R., Jiménez A., Revuelta J.L. (2018). Pathway grafting for polyunsaturated fatty acids production in *Ashbya gossypii* through golden gate rapid assembly. ACS Synth. Biol..

[bib27] Lee M.E., DeLoache W.C., Cervantes B., Dueber J.E. (2015). A highly characterized yeast toolkit for modular, multipart assembly. ACS Synth. Biol..

[bib28] Li M.Z., Elledge S.J. (2007). Harnessing homologous recombination in vitro to generate recombinant DNA via SLIC. Nat. Methods.

[bib29] Lin X., Wang Y., Zhang S., Zhu Z., Zhou Y.J., Yang F., Sun W., Wang X., Zhao Z.K. (2014). Functional integration of multiple genes into the genome of the oleaginous yeast *Rhodosporidium toruloides*. FEMS Yeast Res..

[bib30] Liu Y., Koh C.M.J., Ngoh S. Te, Ji L. (2015). Engineering an efficient and tight d-amino acid-inducible gene expression system in *Rhodosporidium/Rhodotorula* species. Microb. Cell Factories.

[bib31] Liu Y., Yap S.A., Koh C.M.J., Ji L. (2016). Developing a set of strong intronic promoters for robust metabolic engineering in oleaginous *Rhodotorula (Rhodosporidium*) yeast species. Microb. Cell Factories.

[bib32] Liu Z., van den Berg C., Weusthuis R.A., Dragone G., Mussatto S.I. (2021). Strategies for an improved extraction and separation of lipids and carotenoids from oleaginous yeast. Separ. Purif. Technol..

[bib33] Lõoke M., Kristjuhan K., Kristjuhan A. (2011). Extraction of genomic DNA from yeasts for PCR-based applications. Biotechniques.

[bib34] Lopes H.J.S., Bonturi N., Kerkhoven E.J. (2020). C/N ratio and carbon source-dependent lipid production profiling in *Rhodotorula toruloides*. Appl Microbiol Biotechnol.

[bib35] Lopes H.J.S., Bonturi N., Miranda E.A. (2020). *Rhodotorula toruloides* single cell oil production using *eucalyptus urograndis* hemicellulose hydrolysate as a carbon source. Energies.

[bib36] Mata-Gómez L.C., Montañez J.C., Méndez-zavala A., Aguilar C.N. (2018).

[bib37] Ma Y., Liu N., Greisen P., Li J., Qiao K., Huang S., Stephanopoulos G. (2022). Removal of lycopene substrate inhibition enables high carotenoid productivity in *Yarrowia lipolytica*. Nat. Commun..

[bib38] Morin N., Calcas X., Devillers H., Durrens P., Sherman D.J., Nicaud J.M., Neuvéglise C. (2014). Draft genome sequence of *Rhodosporidium toruloides* CECT1137, an oleaginous yeast of biotechnological interest. Genome Announc..

[bib39] Nielsen J., Keasling J.D. (2016). Engineering cellular metabolism. Cell.

[bib40] Nora L.C., Wehrs M., Kim J., Cheng J.-F., Tarver A., Simmons B.A., Magnuson J., Harmon-Smith M., Silva-Rocha R., Gladden J.M., Mukhopadhyay A., Skerker J.M., Kirby J. (2019). A toolset of constitutive promoters for metabolic engineering of *Rhodosporidium toruloides*. Microb. Cell Factories.

[bib41] Oliveira P.M. de, Aborneva D., Bonturi N., Lahtvee P. (2021). Screening and growth characterization of non-conventional yeasts in a hemicellulosic hydrolysate. Front. Bioeng. Biotechnol..

[bib42] Opgenorth P., Costello Z., Okada T., Goyal G., Chen Y., Gin J., Benites V., De Raad M., Northen T.R., Deng K., Deutsch S., Baidoo E.E.K., Petzold C.J., Hillson N.J., Garcia Martin H., Beller H.R. (2019). Lessons from two design-build-test-learn cycles of dodecanol production in Escherichia coli aided by machine learning. ACS Synth. Biol..

[bib43] Otero J.M., Vongsangnak W., Asadollahi M.A., Olivares-Hernandes R., Maury J., Farinelli L., Barlocher L., Østerås M., Schalk M., Clark A., Nielsen J. (2010). Whole genome sequencing of *Saccharomyces cerevisiae*: from genotype to phenotype for improved metabolic engineering applications. BMC Genom..

[bib44] Park Y.K., Nicaud J.M., Ledesma-Amaro R. (2017). The engineering potential of *Rhodosporidium toruloides* as a workhorse for biotechnological applications. Trends Biotechnol..

[bib45] Pinheiro M.J., Bonturi N., Belouah I., Miranda E.A. (2020).

[bib46] Prielhofer R., Barrero J.J., Steuer S., Gassler T., Zahrl R., Baumann K., Sauer M., Mattanovich D., Gasser B., Marx H. (2017). GoldenPiCS: a Golden Gate-derived modular cloning system for applied synthetic biology in the yeast *Pichia pastoris*. BMC Syst. Biol..

[bib47] Quan J., Tian J. (2011). Circular polymerase extension cloning for high-throughput cloning of complex and combinatorial DNA libraries. Nat. Protoc..

[bib48] Rajkumar A.S., Varela J.A., Juergens H., Daran J.-M.G., Morrissey J.P. (2019). Biological parts for *Kluyveromyces marxianus* synthetic biology. Front. Bioeng. Biotechnol..

[bib49] Sambles C., Middelhaufe S., Soanes D., Kolak D., Lux T., Moore K., Matou P., Parker D., Lee R., Love J., Aves S.J. (2017).

[bib50] Sambles C., Middelhaufe S., Soanes D., Kolak D., Lux T., Moore K., Matoušková P., Parker D., Lee R., Love J., Aves S.J. (2017). Genome sequence of the oleaginous yeast *Rhodotorula toruloides* strain CGMCC 2.1609. Genom. Data.

[bib51] Schaub P., Yu Q., Gemmecker S., Poussin-Courmontagne P., Mailliot J., McEwen A.G., Ghisla S., Al-Babili S., Cavarelli J., Beyer P. (2012). On the structure and function of the phytoene desaturase CRTI from pantoea ananatis, a membrane-peripheral and FAD-dependent oxidase/isomerase. PLoS One.

[bib52] Schneider T., Graeff-Hönninger S., French W.T., Hernandez R., Merkt N., Claupein W., Hetrick M., Pham P. (2013). Lipid and carotenoid production by oleaginous red yeast *Rhodotorula glutinis* cultivated on brewery effluents. Energy.

[bib53] Tiukova I.A., Prigent S., Nielsen J., Sandgren M., Kerkhoven E.J. (2019). Genome-scale model of *Rhodotorula toruloides* metabolism. Biotechnol. Bioeng..

[bib54] Tsai Y.-Y., Ohashi T., Kanazawa T., Polburee P., Misaki R., Limtong S., Fujiyama K. (2017). Development of a sufficient and effective procedure for transformation of an oleaginous yeast, *Rhodosporidium toruloides* DMKU3-TK16. Curr. Genet..

[bib55] United Nations General Assembly (2015). Transforming our world: the 2030 agenda for sustainable development. Draft resolution referred to the united Nations summit for the adoption of the post- 2015 development agenda by the general assembly at its sixty-ninth session. UN Doc.

[bib56] Wang G.-Y., Keasling J.D. (2002). Amplification of HMG-CoA reductase production enhances carotenoid accumulation in *Neurospora crassa*. Metab. Eng..

[bib57] Wang Y., Lin X., Zhang S., Sun W., Ma S., Zhao Z.K. (2016). Cloning and evaluation of different constitutive promoters in the oleaginous yeast *Rhodosporidium toruloides*. Yeast.

[bib62] Weber R.W.S., Anke H., Davoli P. (2007). Simple method for the extraction and reversed-phase high-performance liquid chromatographic analysis of carotenoid pigments from red yeasts (*Basidiomycota*. Fungi). J. Chromatogr. A.

[bib58] Weber E., Engler C., Gruetzner R., Werner S., Marillonnet S. (2011). A modular cloning system for standardized assembly of multigene constructs. PLoS One.

[bib59] Xu J., Liu D. (2017). Exploitation of genus Rhodosporidium for microbial lipid production. World J. Microbiol. Biotechnol..

[bib60] Yang F., Zhang S., Tang W., Zhao Z.K., Wiley J. (2008). Identification of the orotidine-5 -monophosphate decarboxylase gene of the oleaginous yeast. Rhodosporidium toruloides.

[bib61] Zhu Z., Zhang S., Liu H., Shen H., Lin X., Yang F., Zhou Y.J., Jin G., Ye M., Zou H., Zhao Z.K. (2012). A multi-omic map of the lipid-producing yeast *Rhodosporidium toruloides*. Nat. Commun..

