# Supplementary material for: Development of a dedicated Golden Gate Assembly Platform (RtGGA) for Rhodotorula toruloides
Source: Metab Eng Commun. 2022 May 23;15:e00200. doi: 10.1016/j.mec.2022.e00200 (PMC9157227; doi:10.1016/j.mec.2022.e00200)
Supplement: Multimedia component 1 [file mmc1.docx]

**
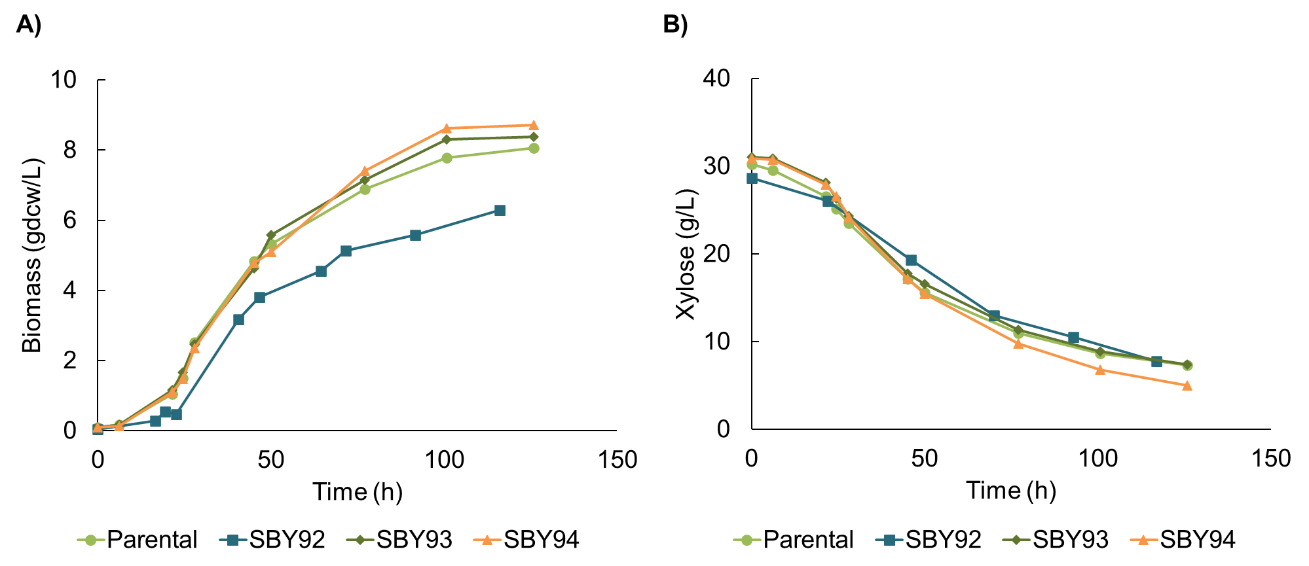
Supplementary Figure S1**. **a)** Growth curve and **b)** xylose consumption profile of strains *R. toruloides* CCT7815 (parental) and SBY92-94. Errors were calculated in terms of standard deviation (n=3) and were ≤15%.

**Supplementary Table 1.** A list of oligonucleotides used for Golden Gate assembly. In the ‘Sequence’ column, the lowercase base pairs are for enzyme anchoring, BsaI recognition site is underlined, and the 4-base pair overhangs are in *italic*.

| Sequence | Purpose |
| --- | --- |
| atttaggtgacactatag | SP6 |
| taatacgactcactatagg | T7 |
| gtaaaacgacggccagt | M13 F |
| caggaaacagctatgac | M13 R |
| gcatggtctca*acgg*tgtccgtattctacatcgacg | Amplification of promoter from XYL1 gene for P1 position |
| atgcggtctca*catt*cgacatggcgtgtattctg |  |
| gcatggtctca*gctt*tgtccgtattctacatcgacg | Amplification of promoter from XYL1 gene for P2 position |
| atgcggtctca*ttgt*cgacatggcgtgtattctg |  |
| gcatggtctca*gtca*tgtccgtattctacatcgacg | Amplification of promoter from XYL1 gene for P3 position |
| atgcggtctca*gtgg*cgacatggcgtgtattctg |  |
| gcatggtctca*acgg*tgtgactgatctggtgttgttctga | Amplification of promoter from GPD1 gene for P1 position |
| atgcggtctca*catt*tggagttcgacgttctcctcgc |  |
| gcatggtctca*gctt*tgtgactgatctggtgttgttctga | Amplification of promoter from GPD gene for P2 position |
| atgcggtctca*ttgt*tggagttcgacgttctcctcgc |  |
| gcatggtctca*gtca*tgtgactgatctggtgttgttctga | Amplification of promoter from GPD gene for P3 position |
| atgcggtctca*gtgg*tggagttcgacgttctcctcgc |  |
| gcatggtctca*acgg*cggctgaggcttccccgacg | Amplification of promoter from ADH2 gene for P1 position |
| atgcggtctca*catt*tgtgactgtcggagacgtggcagc |  |
| gcatggtctca*gctt*cggctgaggcttccccgacg | Amplification of promoter from ADH2 gene for P2 position |
| atgcggtctca*ttgt*tgtgactgtcggagacgtggcagc |  |
| gcatggtctca*gtca*cggctgaggcttccccgacg | Amplification of promoter from ADH2 gene for P3 position |
| atgcggtctca*gtgg*tgtgactgtcggagacgtggcagc |  |
| gcatggtctca*tcta*cgttcaaacatttggcaataaagtttc | Amplification of terminator from NOS gene for T1 position |
| atgcggtctca*aagc*cccgatctagtaacatagatgaca |  |
| gcatggtctca*ggat*cgttcaaacatttggcaataaagttt | Amplification of terminator from NOS gene for T2 position |
| atgcggtctca*tgac*cccgatctagtaacatagatgaca | Amplification of terminator from NOS gene for T2 position |
| gcatggtctca*gtat*cgttcaaacatttggcaataaagttt | Amplification of terminator from NOS gene for T3 position |
| atgcggtctca*actc*cccgatctagtaacatagatgaca |  |
| gcatggtctca*acaa*atgcgcccgcttgcac | Amplification of crtI gene for G2 position |
| cgttaaagatctcgtcaaacagtcgcggg | Site-directed mutagenesis for internal BsaI recognition site removal in crtI gene |
| gtttgacgagatctttaacgatcttggg |  |
| atgcggtctca*atcc*tcaaccgcgcaggtacatc | Amplification of crtI gene for G2 position |
| gcatggtctca*ccac*atgggcggactggactactgg | Amplification of crtYB gene for G3 position |
| atgggaagaccgacggccca | Site-directed mutagenesis for internal BsaI recognition site removal in crtYB gene |
| ccgtcggtcttcccatcctcctc |  |
| atgcggtctca*atac*tcacagcgcctgccacg | Amplification of crtYB gene for G3 position s |
| gcatggtctca*ccac*atgggcaaggagaagacc | Amplification of G418 gene |
| atgcggtctca*atac*ctagaagaactcgtcgagcatgaggt |  |
| gcatggtctca*gtat*cgttcaaacatttggcaataaagttt | Amplification of terminator from NOS gene for Marker |
| atgcggtctca*ccgt*cccgatctagtaacatagatgaca |  |
| acggggtctct*acgg*tactagtagcggccgctg | Amplification of plasmid vector for Marker insertion for Level I construct |
| acggggtctct*acct*ctctagaagcggccgcga |  |
| gcatggtctct*taga*ctctagaagcggccgcga |  |
| atgcggtctca*taga*tcagactttgggaagctcgtgc | Amplification of crte gene for G1 position in Level II constructs |
| gcatggtctca*aatg*tcgctggactggtacgacaac |  |
| gcatggtctca*ggag*ccgcctcctccacctcagcaac | Amplification of upstream insertional regions |
| atgcggtctca*acct*tcatcgtcggcgatgaggaggac |  |
| agagcaggtctcc*tcgg*gatgtggcgaccgggcgcg | Amplification of downstream insertional region |
| atgcggtctca*atgg*gtagaccgtttcgggcgcgac |  |
| actagtagcggccgctgcagggtctcaccattcctgtagtct | Amplification of pGGA for Gibson assembly of pGGA_RFP |
| ctagaagcggccgcgaattcggtctcgctccgtaccaagt |  |
| acttggtacggagcgagaccgaattcgcggccgcttctag | Amplification of RFP for Gibson assembly of pGGA_ RFP |
| actacaggaatggtgagaccctgcagcggccgctactagt |  |
| atgggtccttcaccaccg | Amplification of NAT gene for qPCR verification |
| gagatgaccacgaagcccg |  |
| ctcgtctcgaccgactttgt | Amplification of GAPDH gene for qPCR verification |
| gtaccacgacacgagcttga |  |
| tgatgatggccgcttcg | Amplification of crtE gene for qPCR verification |
| gcccttgttgtttgcgta |  |
| gtccgtcttgaagtacttgg | Amplification of crtI gene for qPCR verification |
| ctccatccctttgtctctgt |  |
| atctccgccgagttgc | Amplification of crtYB gene for qPCR verification |
| gagtaaggcgtgcagtg |  |
| gatgtgggtctcc*aggt*tgtgactgatctggtgttgttctg | Amplification of promoter GPD1 for the characterization cassette |
| gatgtgggtctcc*tgat*tggagttcgacgttctcctcg |  |
| gatgtgggtctcc*aggt*tgtccgtattctacatcga | Amplification of promoter XYL for the characterization cassette |
| gatgtgggtctcc*tgat*cgacatggcgtgtattctg |  |
| gatgtgggtctcc*aggt*cacgcctctgtgactcggta | Amplification of promoter LDP1in for the characterization cassette |
| gatgtgggtctcc*tgat*catggggtagtccgacacct |  |
| gatgtgggtctcc*aggt*cggctgaggcttccccg | Amplification of promoter ADH2 for the characterization cassette |
| gatgtgggtctcc*tgat*tgtgactgtcggagacgtggca |  |
| gatgtgggtctcc*aggt*tccgattctctgatcca | Amplification of promoter FBA for the characterization cassette |
| gatgtgggtctcc*tgat*tgtagctagttagtgttagaag |  |
| atggtgggtctcc*aggt*gcatggccgtctgcc | Amplification of promoter PGI for the characterization cassette |
| atggtgggtctcc*tgat*ggttcgtagcgtggtg |  |
| gatgtgggtctcg*atca*atgggtaccactcttgacg | Amplification of NAT marker for the promoter characterization cassette |
| atgcggtctca*ccgt*cccgatctagtaacatagatgaca |  |
| gcatggtctca*ggag*ccgcctcctccacctcagcaac | Amplification of KU70 upstream insertional sequence for the promoter characterization cassette |
| atgcggtctca*acct*tcatcgtcggcgatgaggaggac |  |
| gcatggtctca*acgg*atgtggcgaccgggcgcgcg | Amplification of KU70 downstream insertional sequence for the promoter characterization cassette |
| atgcggtctca*atgg*gtagaccgtttcgggcgcgac |  |

**Supplementary Table 2.** Comparison between yeast GGA toolkits

| **Author** | YTK (Lee et al., 2015) | GoldenPiCS (Prielhofer et al., 2017) | (Ledesma-Amaro et al., 2018) | (Rajkumar et al., 2019) | (Laroude et al., 2019) | (Bonturi et al., 2022) |
| --- | --- | --- | --- | --- | --- | --- |
| **Micro-organism** | *S. cerevisae* | *P. pastoris* | *A. gossypii* | *K. marxianus* | *Y. lipolytica* | *R. toruloides* |
| **Level 1 (parts)** | Yes | Yes | Yes | Yes | Yes | Yes |
| **Level 1 restriction enzyme** | BsmBi | BsaI | BsaI | BsmBi | BsaI | BsaI |
| **Level 2 (transcriptional unit)** | Yes | Yes | No | Yes | No | Yes |
| **Level 2 restriction enzyme** | BsaI | PbiI | NA | BsaI | NA | BsaI |
| **Level 3 (multigene plasmid)** | Yes | Yes | Yes | Yes | Yes | Yes |
| **Level 3 restriction enzyme** | BsmBi | BsaI | BsaI | BsmBi | BsaI | BsaI |
| **Max. number of tandem TU** | Depends only on assembly connectors availability | 8 | 3 | Depends only on assembly connectors availability | 3 | 3 |
| **Promoter strength validation** | Yes | Yes | No | Yes | Yes | Yes* |
| **Terminator validation** | Yes | Yes | No | Yes | Yes | No |
| **Protein tag validation** | Yes | No | No | No | No | No |
| **Heterologous gene location validation** | Yes | No | No | Yes | Yes | No |
| **Maximum site integration efficiency** | 99.98% | 97% | Not reported | 100% | 45% | Not reported |
| **Endogenous pathway overexpression validation** | No | No | Yes | No | No | Yes |
| **Heterologous pathway overexpression validation** | No | No | Yes | No | Yes | No |
| **CRISPR/Cas9 validation** | Single and multiplex knockout | InDel mutations | No | Single knockout | No | No |
| **Cross compatibility** |  |  |  |  |  | ** |
| **BioBricks compatibility** | Yes | No | No | Yes | No | No |
| * Only one to validate promoter strength by rtPCR. All other use fluorescent protein to do it.  ** Partially compatible | | | | | | |

**Supplementary Table 3.** Comparison between yeast GGA toolkits in terms of overhangs and names of parts

| (Lee et al., 2015) | | (Larroude et al., 2019) | | (Prielhofer et al., 2017) | | (Rajkumar et al., 2019) | | (Ledesma-Amaro et al., 2018) | | (Bonturi et al., 2022) | |
| --- | --- | --- | --- | --- | --- | --- | --- | --- | --- | --- | --- |
| *S. cerevisae* | | *Y. lipolytica* | | *P. pastoris* | | *K. marxianus* | | A. gossypii | | *R. toruloides* | |
| **Name** | **Overhang** | **Name** | **Overhang** | **Name** | **Overhang** | **Name** | **Overhang** | **Name** | **Overhang** | **Name** | **Overhang** |
| 5’ assembly connector | CCCT/AACG | InsUp | GCCT(A)/AGGT(B) | Promoter | GGAG(1)/CATG(2) | Same as YTK | | InsUp | ACAC/ATCA | InsUP | GGAG(P)/AGGT(B) |
| Promoter | AACG/TATG | Marker | AGGT(B)/ACGG(C) | CDS | CATG(2)/GCTT(3) |  |  | Marker | ATCA/GGTC | Marker | AGGT(B)/GTGT(R) |
| Coding sequence | TATG/ATCC | Promoter 1 | ACGG(C)/AATG(D) | Terminator | GCTT(3)/CGCT(4) |  |  | Promoter 1 | GGTC/GATG | P1 | GTGT(R)/AATG(D) |
| Terminator | ATCC/GCTG | Gene 1 | AATG(D)/TCTA(E) | TU1 | GATC(A)/CCGG(B) |  |  | Gene 1 | GATG/AGGG | CDS1 | AATG(D)/TCTA(E) |
| 3’ assembly connector | GCTG/TACA | Terminator 1 | TCTA(E)/GCTT(F) | TU2 | CCGG(B)/AATT(C) |  |  | Terminator 1 | AGGG/CGAT | T1 | TCTA(E)/GCTT(F) |
| Yeast marker | TACA/CTCA | Promoter 2 | GCTT(F)/ACAA(G) | TU3 | AATT(C)/AGCT(D) |  |  | Promoter 2 | CGAT/GTAT | P1 | GCTT(F)/ACAA(G) |
| Plasmid propagation/Chromosomal integration | GAGT/CCGA | Gene 2 | ACAA(G)/GGAT(H) | TU4 | AGCT(D)/AGCT(E) |  |  | Gene 2 | GTAT/TAGT | CDS2 | ACAA(G)/GGAT(H) |
| Plasmid propagation/Chromosomal integration | CCGA/CCCT | Terminator 2 | GGAT(H)/GTCA(I) | TU5 | AGCT(E)/GCTT(F) |  |  | Terminator 2 | TAGT/ACGC | T2 | GGAT(H)/GTCA(I) |
| Multigene | Custom | Promoter 3 | GTCA(I)/CCAC(J) | TU6 | GCTT(F)/CGCT(G) |  |  | Promoter 3 | ACGC/GGAT | P3 | GTCA(I)/CCAC(J) |
|  |  | Gene 3 | CCAC(J)/GTAT(K) | TU7 | CGCT(G)/CAAC(H) |  |  | Gene 3 | GGAT/CTAA | CDS3 | CCAC(J)/GTAT(K) |
|  |  | Terminator 3 | GTAT(K)/GAGT(L) | TU8 | CAAC(H)/TGGT(I) |  |  | Terminator 3 | CTAA/GGCG | T3 | GTAT(K)/TCGG(S) |
|  |  | InsDown | GAGT(L)/TGCG(M) |  |  |  |  | InsDown | GGCG/CTTG | InsD | TCGG(S)/CCAT(Q) |
